# 2,3,4-Trihydroxy­benzaldehyde

**DOI:** 10.1107/S1600536808022241

**Published:** 2008-07-23

**Authors:** Seik Weng Ng

**Affiliations:** a Department of Chemistry, University of Malaya, 50603 Kuala Lumpur, Malaysia

## Abstract

The title compound, C_7_H_6_O_4_, crystallizes with two independent mol­ecules in the asymmetric unit. In both mol­ecules, the 2-hydr­oxy group is bound *via* intra­molecular hydrogen bonds to the aldehyde group. The mol­ecules inter­act through O—H⋯O hydrogen bonds to form a three-dimensional network structure; each hydr­oxy group serves as a donor to only one acceptor atom.

## Related literature

For some references on hydr­oxy-substituted benzaldehydes, see: Kretz *et al.* (2007 ▶); Ng (2005 ▶). For the crystal structures of Schiff base derivatives of 2,3,4-trihydroxy­salicylaldehyde, see: Petek *et al.* (2006 ▶); Sun *et al.* (2007 ▶).
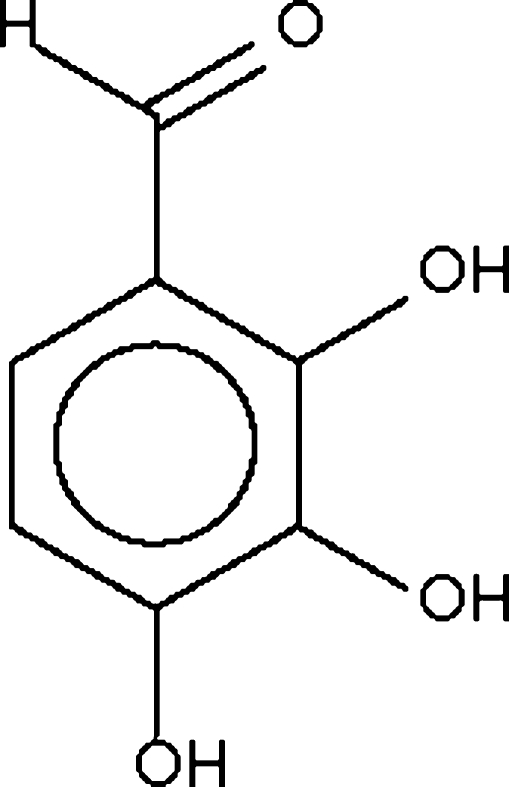


## Experimental

### 

#### Crystal data

C_7_H_6_O_4_
*M* *_r_* = 154.12
Monoclinic, 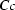

*a* = 3.6222 (3) Å
*b* = 24.006 (2) Å
*c* = 14.8965 (9) Å
β = 93.524 (5)°
*V* = 1292.9 (2) Å^3^
*Z* = 8
Mo *K*α radiation
μ = 0.13 mm^−1^
*T* = 100 (2) K
0.30 × 0.03 × 0.03 mm

#### Data collection

Bruker SMART APEX diffractometer
Absorption correction: none
5464 measured reflections
1491 independent reflections
1087 reflections with *I* > 2σ(*I*)
*R*_int_ = 0.088

#### Refinement

*R*[*F* ^2^ > 2σ(*F* ^2^)] = 0.054
*wR*(*F* ^2^) = 0.132
*S* = 1.01
1491 reflections
217 parameters
8 restraints
H atoms treated by a mixture of independent and constrained refinement
Δρ_max_ = 0.30 e Å^−3^
Δρ_min_ = −0.32 e Å^−3^

### 

Data collection: *APEX2* (Bruker, 2007 ▶); cell refinement: *SAINT* (Bruker, 2007 ▶); data reduction: *SAINT*; program(s) used to solve structure: *SHELXS97* (Sheldrick, 2008 ▶); program(s) used to refine structure: *SHELXL97* (Sheldrick, 2008 ▶); molecular graphics: *X-SEED* (Barbour, 2001 ▶); software used to prepare material for publication: *publCIF* (Westrip, 2008 ▶).

## Supplementary Material

Crystal structure: contains datablocks global, I. DOI: 10.1107/S1600536808022241/bt2748sup1.cif

Structure factors: contains datablocks I. DOI: 10.1107/S1600536808022241/bt2748Isup2.hkl

Additional supplementary materials:  crystallographic information; 3D view; checkCIF report

## Figures and Tables

**Table 1:** Hydrogen-bond geometry (Å, °)

*D*—H⋯*A*	*D*—H	H⋯*A*	*D*⋯*A*	*D*—H⋯*A*
O2—H2⋯O1	0.84 (1)	1.99 (5)	2.631 (5)	133 (6)
O3—H3⋯O7^i^	0.84 (1)	2.04 (3)	2.816 (5)	153 (6)
O4—H4⋯O1^ii^	0.84 (1)	1.90 (3)	2.701 (5)	159 (6)
O6—H6⋯O5	0.84 (1)	1.87 (3)	2.653 (5)	154 (6)
O7—H7⋯O2	0.84 (1)	2.02 (3)	2.772 (5)	149 (6)
O8—H8⋯O5^iii^	0.84 (1)	1.86 (2)	2.679 (5)	162 (7)

Symmetry codes: (i) 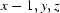; (ii) 
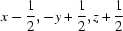
; (iii) 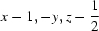.

**Fig. 1.**
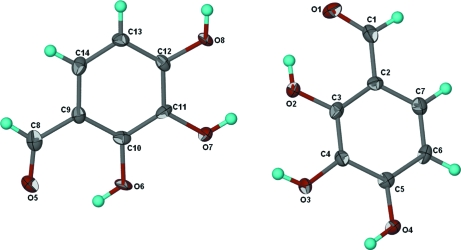
Plot (Barbour, 2001) of the two independent molecules of 2,3,4-trihydroxybenzaldehyde with 50% probability ellipsoids. Hydrogen atoms are drawn as spheres of arbitrary radius.

## supplementary crystallographic information

### Comment

2,3,4-Trihydroxybenzaldehyde condenses with primary amines to afford Schiff bases. The crystal structures of only few such Schiff bases have been reported. The 2-fluoroaniline derivative exists as a zwitterion as the hydrogen atom of the 2-hydroxy substituent is transferred to the imino nitrogen atom (Petek *et al.*, 2006). On the other hand, the antipyrine derivative has the expected neutral structure (Sun *et al.*, 2007). Although there are several structural studies on hydroxy-substituted benzaldehydes (Kretz *et al.*, 2007; Ng, 2005), the structure of 2,3,4-trihydroxybenzaldehyde has not been reported.

2,3,4-Trihydroxybenzaldehyde (Scheme I) crystallizes with two independent molecules in the asymmetric unit. In both, the 2-hydroxy group is hydrogen-bonded to the aldehyde group via an intramolecular hydrogen bond (Fig. 1). The molecules interact through O–H···O hydrogen bonds to form a three-dimensional network structure; each hydroxy group serves as donor to only one acceptor atom.

### Experimental

Commercially available 2,3,4-trihydroxybenzaldehyde was recrystallized from ethanol to furnished light-brown, needled-shaped crystals.

### Refinement

Carbon-bound H-atoms were placed in calculated positions (C—H 0.95 Å) and were included in the refinement in the riding model approximation, with *U*(H) set to 1.2*U*_eq_(C). The hydroxy H-atoms were located in a difference Fourier map, and were refined with a distance restraint of O–H 0.84±0.01 Å; their displacement parameters were set to 1.5*U*_eq_(O).

### Crystal data

**Table:**

C_7_H_6_O_4_	*F*_000_ = 640
*M**_r_* = 154.12	*D*_x_ = 1.584 Mg m^−^^3^
Monoclinic, *C**c*	Mo *K*α radiation λ = 0.71073 Å
Hall symbol: C c	Cell parameters from 446 reflections
*a* = 3.6222 (3) Å	θ = 2.8–19.5º
*b* = 24.006 (2) Å	µ = 0.13 mm^−^^1^
*c* = 14.8965 (9) Å	*T* = 100 (2) K
β = 93.524 (5)º	Needle, light brown
*V* = 1292.9 (2) Å^3^	0.30 × 0.03 × 0.03 mm
*Z* = 8	

### Data collection

**Table:**

Bruker SMART APEX diffractometer	1087 reflections with *I* > 2σ(*I*)
Radiation source: fine-focus sealed tube	*R*_int_ = 0.088
Monochromator: graphite	θ_max_ = 27.5º
*T* = 100(2) K	θ_min_ = 1.7º
ω scans	*h* = −4→3
Absorption correction: None	*k* = −30→30
5464 measured reflections	*l* = −19→19
1491 independent reflections	

### Refinement

**Table:**

Refinement on *F*^2^	Secondary atom site location: difference Fourier map
Least-squares matrix: full	Hydrogen site location: inferred from neighbouring sites
*R*[*F*^2^ > 2σ(*F*^2^)] = 0.054	H atoms treated by a mixture of independent and constrained refinement
*wR*(*F*^2^) = 0.132	*w* = 1/[σ^2^(*F*_o_^2^) + (0.0467*P*)^2^ + 2.3366*P*] where *P* = (*F*_o_^2^ + 2*F*_c_^2^)/3
*S* = 1.01	(Δ/σ)_max_ = 0.001
1491 reflections	Δρ_max_ = 0.30 e Å^−^^3^
217 parameters	Δρ_min_ = −0.32 e Å^−^^3^
8 restraints	Extinction correction: none
Primary atom site location: structure-invariant direct methods	Absolute structure: Friedel pairs were merged

### Fractional atomic coordinates and isotropic or equivalent isotropic displacement parameters (Å^2^)

**Table:**

	*x*	*y*	*z*	*U*_iso_*/*U*_eq_	
O1	0.5000 (11)	0.19999 (15)	0.5000 (3)	0.0203 (9)	
O2	0.4561 (11)	0.18738 (14)	0.6744 (3)	0.0177 (9)	
H2	0.432 (19)	0.172 (2)	0.6236 (19)	0.027*	
O3	0.2999 (11)	0.23611 (14)	0.8356 (2)	0.0186 (9)	
H3	0.253 (18)	0.2022 (8)	0.827 (4)	0.028*	
O4	0.0704 (11)	0.34509 (15)	0.8358 (3)	0.0187 (9)	
H4	0.046 (19)	0.324 (2)	0.879 (3)	0.028*	
O5	1.4505 (11)	−0.00937 (16)	1.0289 (3)	0.0206 (9)	
O6	1.1992 (11)	0.08512 (14)	0.9559 (2)	0.0195 (9)	
H6	1.297 (17)	0.062 (2)	0.993 (3)	0.029*	
O7	0.8968 (11)	0.13655 (14)	0.8094 (3)	0.0187 (9)	
H7	0.801 (18)	0.142 (2)	0.7575 (19)	0.028*	
O8	0.7396 (10)	0.07867 (14)	0.6555 (2)	0.0172 (9)	
H8	0.696 (19)	0.056 (2)	0.613 (3)	0.026*	
C1	0.3888 (16)	0.2489 (2)	0.5099 (4)	0.0182 (12)	
H1	0.3571	0.2717	0.4579	0.022*	
C2	0.3068 (15)	0.2725 (2)	0.5942 (4)	0.0140 (11)	
C3	0.3431 (15)	0.2416 (2)	0.6752 (4)	0.0148 (11)	
C4	0.2636 (16)	0.2646 (2)	0.7557 (4)	0.0151 (12)	
C5	0.1496 (15)	0.3204 (2)	0.7578 (4)	0.0153 (11)	
C6	0.1124 (15)	0.3520 (2)	0.6789 (4)	0.0171 (12)	
H6A	0.0328	0.3896	0.6812	0.021*	
C7	0.1914 (16)	0.3285 (2)	0.5982 (4)	0.0184 (12)	
H7A	0.1680	0.3501	0.5448	0.022*	
C8	1.3595 (16)	−0.0319 (2)	0.9554 (4)	0.0182 (12)	
H8A	1.4039	−0.0707	0.9498	0.022*	
C9	1.1938 (15)	−0.0035 (2)	0.8791 (4)	0.0146 (11)	
C10	1.1232 (16)	0.0546 (2)	0.8814 (4)	0.0150 (11)	
C11	0.9686 (16)	0.08085 (19)	0.8055 (4)	0.0142 (11)	
C12	0.8902 (15)	0.0499 (2)	0.7274 (3)	0.0142 (11)	
C13	0.9584 (15)	−0.0072 (2)	0.7239 (4)	0.0160 (12)	
H13	0.9043	−0.0276	0.6701	0.019*	
C14	1.1054 (15)	−0.0333 (2)	0.7999 (4)	0.0167 (12)	
H14	1.1480	−0.0723	0.7988	0.020*	

### Atomic displacement parameters (Å^2^)

**Table:**

	*U*^11^	*U*^22^	*U*^33^	*U*^12^	*U*^13^	*U*^23^
O1	0.023 (2)	0.0189 (19)	0.019 (2)	0.0023 (16)	0.0050 (18)	−0.0053 (15)
O2	0.025 (2)	0.0122 (17)	0.015 (2)	0.0033 (16)	−0.0038 (18)	−0.0012 (15)
O3	0.032 (3)	0.0111 (17)	0.013 (2)	0.0021 (16)	0.0006 (18)	0.0028 (14)
O4	0.027 (2)	0.0147 (17)	0.015 (2)	0.0041 (17)	0.0023 (17)	−0.0010 (15)
O5	0.023 (2)	0.023 (2)	0.015 (2)	0.0024 (17)	−0.0034 (17)	0.0012 (16)
O6	0.032 (3)	0.0147 (18)	0.011 (2)	−0.0003 (16)	−0.0070 (18)	−0.0039 (15)
O7	0.029 (3)	0.0124 (16)	0.0140 (18)	0.0040 (16)	−0.0032 (17)	−0.0030 (15)
O8	0.023 (2)	0.0163 (18)	0.011 (2)	0.0023 (16)	−0.0065 (17)	−0.0020 (14)
C1	0.017 (3)	0.020 (3)	0.017 (3)	0.001 (2)	−0.005 (3)	0.003 (2)
C2	0.010 (3)	0.018 (2)	0.014 (3)	0.001 (2)	0.000 (2)	−0.003 (2)
C3	0.007 (3)	0.015 (2)	0.022 (3)	0.002 (2)	−0.002 (2)	−0.002 (2)
C4	0.016 (3)	0.014 (2)	0.015 (3)	0.001 (2)	−0.002 (2)	0.001 (2)
C5	0.011 (3)	0.017 (3)	0.019 (3)	−0.001 (2)	0.003 (2)	−0.001 (2)
C6	0.014 (3)	0.012 (2)	0.025 (3)	0.002 (2)	0.000 (3)	0.000 (2)
C7	0.021 (3)	0.017 (3)	0.017 (3)	0.002 (2)	−0.001 (3)	0.003 (2)
C8	0.014 (3)	0.019 (3)	0.022 (3)	0.000 (2)	0.002 (2)	0.006 (2)
C9	0.012 (3)	0.015 (2)	0.016 (3)	0.000 (2)	0.001 (2)	0.001 (2)
C10	0.011 (3)	0.021 (3)	0.014 (3)	−0.002 (2)	0.000 (2)	−0.004 (2)
C11	0.013 (3)	0.012 (2)	0.017 (3)	0.000 (2)	0.001 (2)	−0.005 (2)
C12	0.009 (3)	0.020 (2)	0.014 (3)	0.000 (2)	0.000 (2)	0.003 (2)
C13	0.013 (3)	0.020 (3)	0.014 (3)	0.000 (2)	−0.002 (2)	−0.004 (2)
C14	0.012 (3)	0.014 (3)	0.024 (3)	0.001 (2)	−0.001 (2)	0.000 (2)

### Geometric parameters (Å, °)

**Table:**

O1—C1	1.253 (6)	C9—C10	1.421 (7)
O2—C3	1.365 (6)	C10—C11	1.382 (8)
O3—C4	1.372 (6)	C11—C12	1.395 (7)
O4—C5	1.351 (6)	C12—C13	1.394 (7)
O5—C8	1.248 (7)	C13—C14	1.372 (7)
O6—C10	1.343 (6)	O2—H2	0.84 (1)
O7—C11	1.364 (6)	O3—H3	0.84 (1)
O8—C12	1.361 (6)	O4—H4	0.84 (1)
C1—C2	1.426 (7)	O6—H6	0.84 (1)
C2—C7	1.409 (7)	O7—H7	0.84 (1)
C2—C3	1.416 (7)	O8—H8	0.84 (1)
C3—C4	1.367 (7)	C1—H1	0.9500
C4—C5	1.402 (7)	C6—H6A	0.9500
C5—C6	1.398 (7)	C7—H7A	0.9500
C6—C7	1.374 (7)	C8—H8A	0.9500
C8—C9	1.425 (8)	C13—H13	0.9500
C9—C14	1.398 (7)	C14—H14	0.9500
			
C3—O2—H2	114 (4)	C10—C9—C8	121.1 (5)
C4—O3—H3	110 (5)	O6—C10—C11	118.6 (4)
C5—O4—H4	116 (5)	O6—C10—C9	121.9 (5)
C10—O6—H6	104 (4)	C11—C10—C9	119.5 (5)
C11—O7—H7	101 (4)	O7—C11—C10	118.7 (5)
C12—O8—H8	108 (4)	O7—C11—C12	121.9 (5)
O1—C1—C2	124.2 (5)	C10—C11—C12	119.4 (4)
C7—C2—C3	118.4 (5)	O8—C12—C13	122.3 (5)
C7—C2—C1	119.7 (5)	O8—C12—C11	115.9 (4)
C3—C2—C1	121.9 (4)	C13—C12—C11	121.9 (5)
O2—C3—C4	118.2 (5)	C14—C13—C12	118.6 (5)
O2—C3—C2	120.4 (5)	C13—C14—C9	121.3 (5)
C4—C3—C2	121.5 (4)	O1—C1—H1	117.9
C3—C4—O3	123.1 (4)	C2—C1—H1	117.9
C3—C4—C5	118.9 (5)	C7—C6—H6A	120.1
O3—C4—C5	118.0 (5)	C5—C6—H6A	120.1
O4—C5—C6	118.0 (5)	C6—C7—H7A	119.8
O4—C5—C4	121.1 (5)	C2—C7—H7A	119.8
C6—C5—C4	120.9 (5)	O5—C8—H8A	117.6
C7—C6—C5	119.9 (5)	C9—C8—H8A	117.6
C6—C7—C2	120.4 (5)	C14—C13—H13	120.7
O5—C8—C9	124.7 (5)	C12—C13—H13	120.7
C14—C9—C10	119.4 (5)	C13—C14—H14	119.3
C14—C9—C8	119.5 (5)	C9—C14—H14	119.3

### Hydrogen-bond geometry (Å, °)

**Table:**

*D*—H···*A*	*D*—H	H···*A*	*D*···*A*	*D*—H···*A*
O2—H2···O1	0.84 (1)	1.99 (5)	2.631 (5)	133 (6)
O3—H3···O7^i^	0.84 (1)	2.04 (3)	2.816 (5)	153 (6)
O4—H4···O1^ii^	0.84 (1)	1.90 (3)	2.701 (5)	159 (6)
O6—H6···O5	0.84 (1)	1.87 (3)	2.653 (5)	154 (6)
O7—H7···O2	0.84 (1)	2.02 (3)	2.772 (5)	149 (6)
O8—H8···O5^iii^	0.84 (1)	1.86 (2)	2.679 (5)	162 (7)

Symmetry codes: (i) *x*−1, *y*, *z*; (ii) *x*−1/2, −*y*+1/2, *z*+1/2; (iii) *x*−1, −*y*, *z*−1/2.
